# Association between *Helicobacter pylori* infection and serum thyroid stimulating hormone in the National Health and Nutrition Examination Survey 1999-2000

**DOI:** 10.3389/fendo.2022.1018267

**Published:** 2022-11-11

**Authors:** Jinyun Wang, Dingwei Liu, Yong Xie

**Affiliations:** ^1^ Department of Gastroenterology, Digestive Disease Hospital, The First Affiliated Hospital of Nanchang University, Nanchang, Jiangxi, China; ^2^ Department of Gastroenterology, The First Affiliated Hospital of Nanchang University, Nanchang, Jiangxi, China; ^3^ Department of Gastroenterology, JiangXi Clinical Research Center for Gastroenterology, The First Affiliated Hospital of Nanchang University, Nanchang, China

**Keywords:** thyroid stimulating hormone, TSH, *Helicobacter pylori* infection, NHANES, CDC

## Abstract

**Objective:**

Current evidence on the associations between plasma thyroid stimulating hormone and *Helicobacter pylori* infection is conflicting. Therefore, our study aimed to examine TSH in relation to *H. pylori* infection.

**Methods:**

Based on the US National Health and Nutrition Examination Survey (NHANES) 1999-2000, a cross-sectional study was conducted with 948 participants aged 30 to 85 years. The associations between *H. pylori* seropositivity and TSH were evaluated using binary logistic regression models. A subgroup analysis stratified by sex, age, and body mass index was conducted.

**Results:**

A higher serum TSH level was found in subjects with *H. pylori* seropositive than in subjects with *H. pylori* seronegative. A significant positive association was found between *H. pylori* seropositivity and TSH with increasing quartiles of hormonal levels in univariate regression models (Q4 vs Q1: OR = 1.659; 95% CI, 1.152-2.389) and in multivariate regression models (Q4 vs Q1: OR = 1.604; 95% CI, 1.087-2.367). In stratified analyses, the adjusted association of serum TSH with *H. pylori* seropositivity was statistically significant in male (Q4 vs Q1: OR = 1.894; 95% CI, 1.109-3.235), normal BMI (Q4 vs Q1: OR = 1.894; 95% CI, 1.109-3.235), overweight (Q4 vs Q1: OR = 2.124; 95% CI, 1.047-4.308);, obese (Q4 vs Q1: OR = 0.429; 95% CI, 0.220-0.837), and age over 60 years (Q4 vs Q1: OR = 1.999; 95% CI, 1.118-3.575).

**Conclusion:**

High TSH levels were associated with *H. pylori* infection, especially among male, overweight and elderly adults.

## 1 Introduction

*Helicobacter pylori*, a gastric pathogen infecting more than half of the world’s population, which leads to chronic inflammation of the gastric mucosa (1). *H. pylori* infection has been associated with many gastric diseases, including chronic gastritis, peptic ulcers, gastric cancer, and mucosa-associated lymphoid tissue lymphoma (2). Furthermore, *H. pylori* infection has been proven to be associated with many extra-gastric diseases, such as autoimmune thyroid diseases, metabolic, neurological, and cardiovascular diseases (3–8).

A high level of thyroid stimulating hormone can negatively affect metabolic health in the euthyroid state (9). Previous studies have examined the role of *H. pylori* in thyroid disease (10). Silva et al. found that *H. pylori* Infection is associated with thyroid dysfunction in children with congenital hypothyroidism (11). Bugdaci et al. found that patients with hypothyroidism could not be achieved normal thyrotropin levels despite treatment with high doses of thyroxine under the condition of *H. pylori* infection (12). In addition, the association between *H. pylori* infection and treatment-refractory hypothyroidism has been proven (13, 14). TSH reflects both hypothyroidism and subclinical hyperthyroidism as a sensitive marker of thyroid function (15).However, the relationship between *H. pylori* infection and plasma TSH in the general population is limited and controversial (16–19).

In this study, we evaluated the association between *H. pylori* seroprevalence and plasma TSH level based on data from the 1999–2000 National Health and Nutrition Examination Survey (NHANES). Previous studies have reported that age (20), sex (21), race (22, 23), BMI (24), waist circumference (25), household size (26), education level (27), smoking behavior (25), alcohol behavior (28)and serum C-reactive protein (29) may were associated with *H. pylori* infection, so this study includes these variables for analysis.

## 2 Materials and methods

### 2.1 Study design

NHANES is a representative survey of the general US population, providing a wide range of information about the health and nutrition of the general population, and utilizing a complex, multistage, and probability sampling design to provide information about nutrition and health for the general US population (30, 31). Study data were collected from the US National Health and Nutrition Examination Survey (NHANES) 1999–2000. Because participants of this cycle included both *H. pylori* infection and plasma thyroid stimulating hormone data.

### 2.2 Sample size

Sample size was calculated assuming the following parameters: alpha error = 0.05, power = 80%, expected effect size: odds ratio (OR) = 1.5 (for the TSH as a risk factor), prevalence of *H. pylori* (outcome) = 0.50. A total of 150 participants were needed in the study. Considering the influence of other confounding factors, we expand the sample size as much as possible.

### 2.3 Study participants, inclusion criteria and exclusion criteria

**Figure 1:**
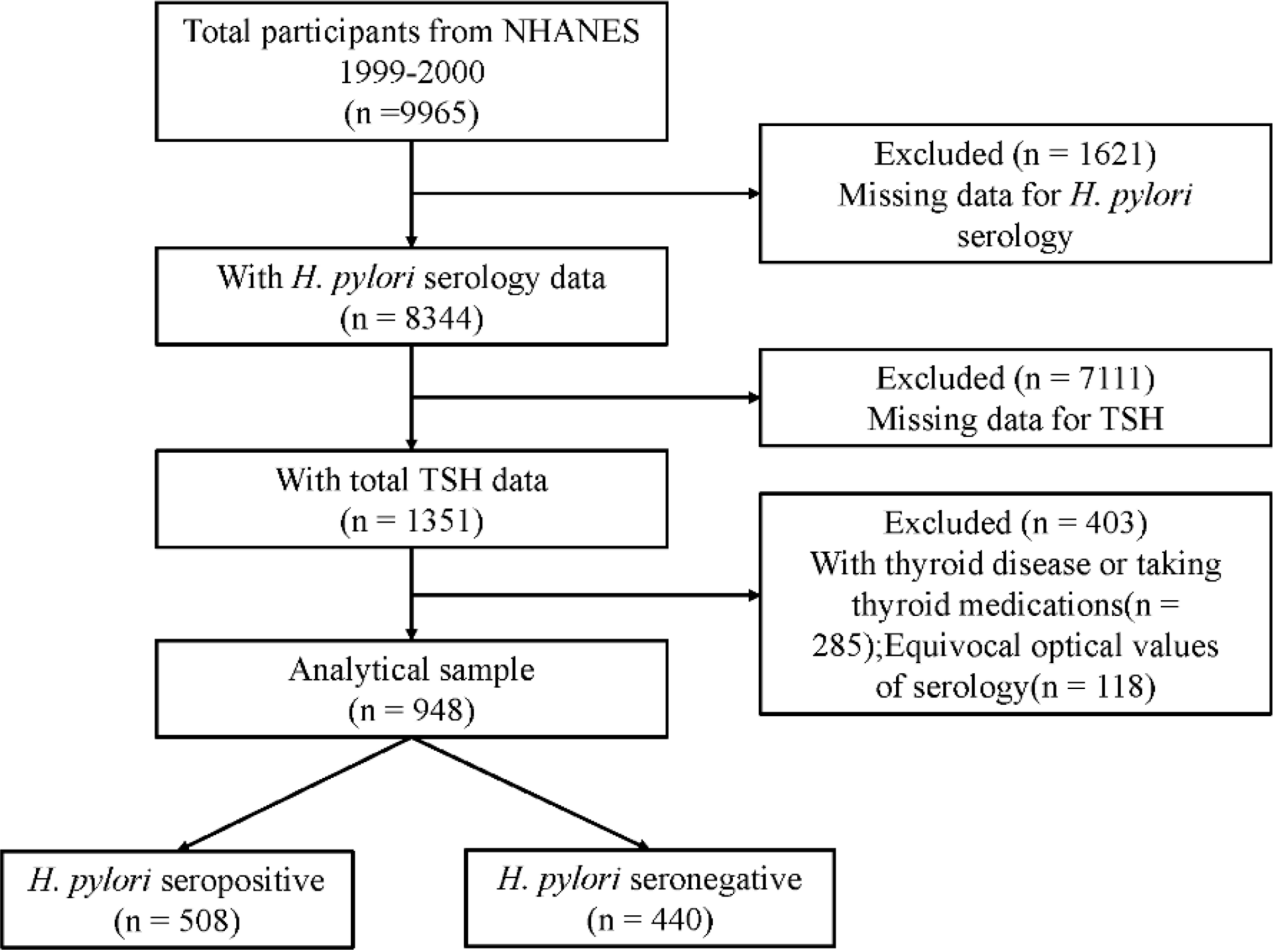
The sample selection flow chart.

In NHANES 1999–2000 cycle, 9965 subjects participated. After excluding subjects without information on laboratory and demographic variables, individuals with thyroid disease and those taking thyroid medications (32). 948 subjects were finally included for analyses. The sample selection flow chart is presented in **Figure 1**.

### 2.4 *Helicobacter pylori* status

NHANES assessed *H. pylori* exposure by IgG antibody detection using an enzyme-linked immunoassay(ELISA) (33). Raw IgG results were not published as part of the NHANES datasets. The sensitivity, specificity, and reproducibility of ELISA were comparable to other serological tests for antibodies, such as immunofluorescence, complement fixation, hemagglutination, and radioimmunoassays (34, 35). Standard ELISA cut-offs were used to categorize participants into seropositive (optical density (OD) value ≥1.1) or seronegative (OD value <0.9) to *H. pylori (* 36). Equivocal values (0.9–1.1) were excluded to prevent misleading statistical outcomes in this study (31).

### 2.5 Thyroid stimulating hormone

In this study, the dependent variable was *H. pylori* seropositivity, and the targeted independent variable was plasma thyroid stimulating hormone(TSH). The measurements of TSH were determined by the IMx ultrasensitive hTSH II microparticle enzyme immunoassay technique (37). serum TSH were obtained from the NHANES laboratory data files. Thyroid medications were identified in the prescription drug medication document.

### 2.6 Covariates

In this study, we added several covariates based on previous studies (31, 35, 36, 38), finally including age, sex, race, BMI, waist circumference, household size, education level, smoking behavior, alcohol behavior and serum C reactive protein. For covariates, sex, race, educational level, BMI, household size, education level, alcohol behavior and smoking behavior were used as categorical variables; age, waist circumference and serum C reactive protein were used as continuous variables. More detailed information on *H. pylori* seropositivity, serum TSH, and the covariates is publicly available at http://www.cdc.gov/nchs/nhanes/.

### 2.7 Statistical analyses

Continuous variables are presented as mean ± SD, and categorical variables are reported as numbers and percentages (15). The baseline characteristics among the different groups were compared using Chi-square test, Student’s t test, and Fisher’s exact test, as appropriate (15). The TSH levels were stratified into quartiles (Q1 to Q4). Subsequently, logistic regression models were used to assess the independent association between *H. pylori* seropositivity and TSH levels. In model 1, we adjusted no factors. In model 2, we adjusted age, sex, and BMI. Model 3 was additionally adjusted for race, educational level and household size. Differences with P < 0.05 were considered statistically significant. Finally, we stratified the above analyses by potential effect modifiers, including age groups, sex and BMI. In addition, the regression analyses were reported as ORs and 95% CIs. All statistical analyses were performed using SPSS version 26.

## 3 Results

### 3.1 Characteristics of included subjects

**Figure 2:**
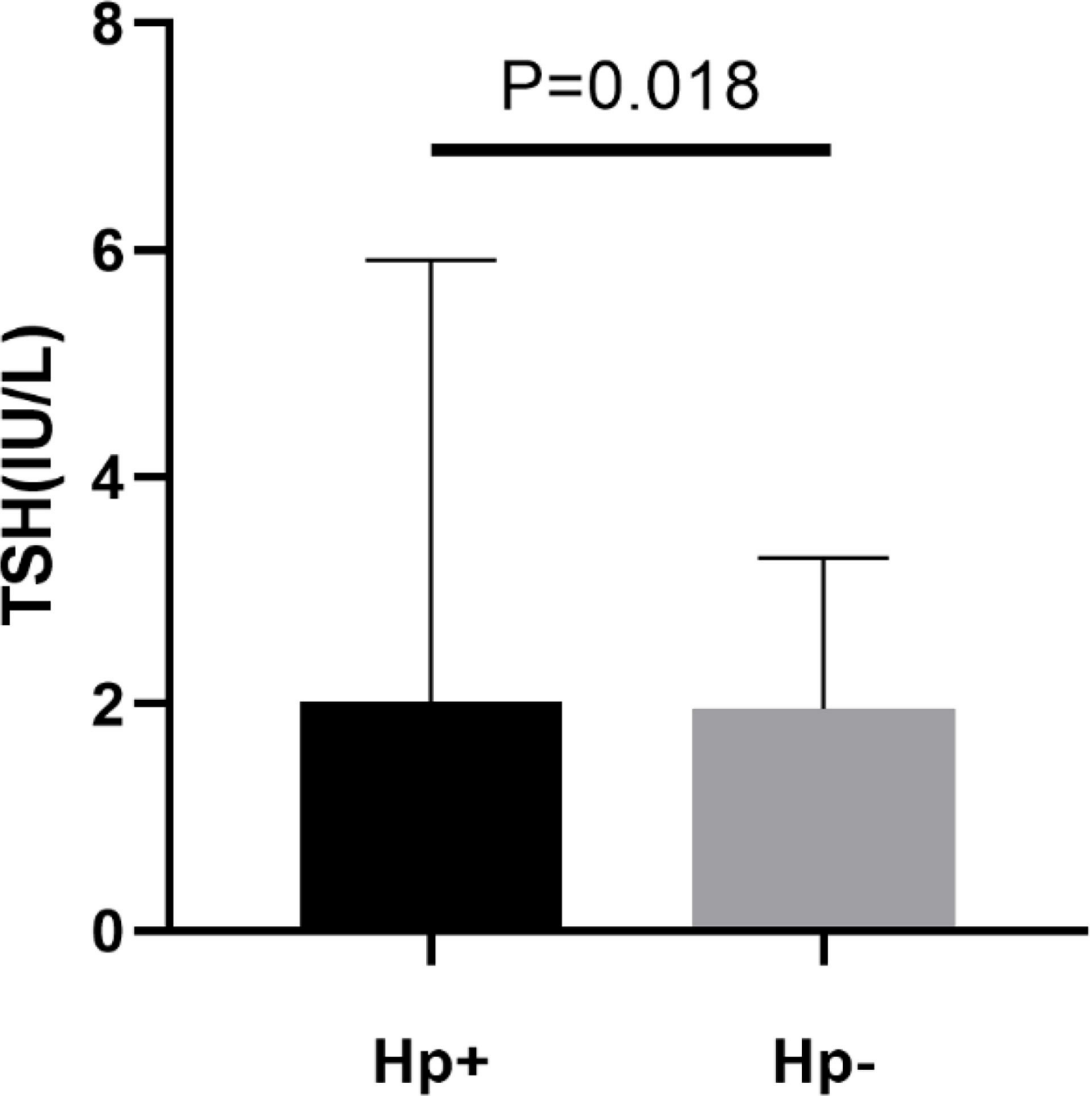
Levels of plasma thyroid stimulating hormone (TSH) in patients with *H. pylori* seropositive (Hp+) and *H. pylori* seronegative (Hp-).

A total of 948 subjects were included in final analyses, of which 508 (53.59%) subjects were *H. pylori* seropositive and 440 (46.41%) subjects were *H. pylori* seronegative. In these two groups, age, race, household size, educational level and TSH were significantly different (P < 0.05). Compared with *H. pylori* seronegative individuals, patients with *H. pylori* seropositive had higher TSH levels (mean TSH, 2.02 IU/L vs 1.96 IU/L). More details are presented in **Table 1** and **Figure 2**.

**Table 1:** Weighted characteristics of included subjects.

	*H. pylori* seropositive (n = 508)	*H. pylori* seronegative (n = 440)	T/X^2^	*P* value
Age (years)	56.25(15.70)	54.18(16.30)	1.989	**0.039**
Sex			3.277	0.070
Male	262(51.57%)	201(45.68%)		
Female	246(48.43%)	239(54.32%)		
Race			182.352	**<0.001**
Non-Hispanic white	200(39.37%)	53(12.05%)		
Non-Hispanic black	17(3.35%)	10(2.27%)		
Mexican American	137(26.97%)	307(60.77%)		
Other Hispanic	114(22.44%)	59(13.41%)		
Other races	40(7.87%)	11(11.5%)		
BMI (kg/m^2^)			2.878	0.090
Undernutrition(<18)	3(0.59%)	3(0.68%)		
Normal (18-25)	131(25.79%)	139(31.59%)		
Overweight (25-30)	207(40.75%)	157(35.68%)		
Obese (30+)	167(32.87%)	141(32.05%)		
Waist circumference(cm)	97.81(14.32)	98.04(15.37)	-0.241	0.874
Household size			10.495	**0.001**
Small (1-3members)	313(61.61%)	315(71.59%)		
Large (4 or more members)	195(38.39%)	125(28.41%)		
Educational level			65.687	**<0.001**
Less than high school	378(74.41%)	215(48.86%)		
High school or above	130(25.59%)	310(51.14%)		
Smoking behavior			1.412	0.570
Never	412(81.10%)	363(82.50%)		
Sometimes	13(2.56%)	15(3.41%)		
Everyday	83(16.34%)	62(14.09%)		
Alcohol behavior			0.680	0.124
Yes	85(16.73%)	65(14.77%)		
No	334(83.27%)	389(85.23%)		
CRP(mg/dL)	0.55(1.12)	0.49(0.92)	0.820	0.098
Serum TSH level(IU/L)	2.02(3.89)	1.96(1.33)	0.306	**0.018**

Other race/ethnicity includes all race/ethnicity other than Mexico-American, non-Hispanic white and black. CRP, C reactive protein; BMI, Body mass index; TSH, thyroid stimulating hormone. T value is for continuous variables and X^2^ Value is for categorical variables. Bold represents statistically significant.

### 3.2 Association between *H. pylori* Seropositivity and TSH

#### 3.2.1 Multiple regression model

Three weighted univariate and multivariate regression models were constructed: model 1, unadjusted; model 2, age, sex, and BMI that were adjusted; and model 3, covariates presented in **Table 2** that were adjusted. In the unadjusted model, a significant positive association was found between *H. pylori* seropositivity and TSH with increasing quartiles of hormonal levels (Q4 vs Q1: OR = 1.659; 95% CI, 1.152-2.389; P=0.006). Furthermore, this association still existed after adjusting for confounding factors in the model 2 (Q4 vs Q1: OR = 1.794; 95% CI, 1.236-2.605; P=0.002) and model 3 (Q4 vs Q1: OR = 1.604; 95% CI, 1.087-2.367; P=0.017). Details are presented in **Table 2**.

**Table 2:** Association of thyroid stimulating hormone level with *Helicobacter pylori* seropositivity.

TSH	Model 1		Model 2		Model 3	
	OR (95% CI)	P value	OR (95% CI)	P value	OR (95% CI)	P value
Q1	Ref		Ref		Ref	
Q2	1.345 (0.935-1.936)	0.110	1.390 (0.963-2.008)	0.079	1.369 (0.933-2.009)	0.108
Q3	1.266 (0.878-1.824)	0.207	1.326 (0.915-1.921)	0.136	1.255 (0.853-1.846)	0.249
Q4	1.659 (1.152-2.389)	0.006	1.794 (1.236-2.605)	0.002	1.604 (1.087-2.367)	0.017

Model 1: no covariates were adjusted; model 2: age, sex, and body mass index were adjusted; model 3: age, sex, body mass index, race, educational level and household size were adjusted. OR: Odds Ratio. Q1:<1.09 mIU/L, Q2: 1.09-1.57 mIU/L, Q3: 1.57-2.295 mIU/L, Q4:>2.295 mIU/L.

#### 3.2.2 Subgroup analyses

In the subgroup analyses stratified by sex, a positive association was observed between the *H. pylori* seropositivity and TSH in male (Q4 vs Q1: OR = 1.894; 95% CI, 1.109-3.235; P=0.019). However, this association disappeared after adjusting for confounding factors in the multivariate regression model (Q4 vs Q1: OR = 1.659; 95% CI, 0.942-2.920; P=0.080). The TSH was not related to *H. pylori* seropositivity in female. Details are presented in **Table 3**.

**Table 3:** Association of thyroid stimulating hormone level with Helicobacter pylori seropositivity based on subgroup of sex.

Subgroup	TSH	Model 1		Model 2		Model 3	
		OR (95% CI)	P value	OR (95% CI)	P value	OR (95% CI)	P value
**Male**	Q1	Ref		Ref		Ref	
	Q2	1.658(0.982-2.798)	0.058	1.660(0.982-2.805)	0.059	1.686(0.969-2.933)	0.064
	Q3	1.540(0.899-2.637)	0.116	1.527(0.887-2.628)	0.127	1.377(0.777-2.442)	0.273
	Q4	1.915(1.123-3.265)	0.017	1.894(1.109-3.235)	0.019	1.659(0.942-2.920)	0.08
**Female**	Q1	Ref		Ref		Ref	
	Q2	1.122(0.672-1.873)	0.66	1.170(0.693-1.976)	0.556	1.126(0.655-1.935)	0.667
	Q3	1.056(0.639-1.746)	0.83	1.110(0.665-1.855)	0.689	1.088(0.641-1.848)	0.754
	Q4	1.456(0.880-2.409)	0.143	1.608(0.961-2.692)	0.071	1.451(0.851-2.475)	0.171

Model 1: no covariates were adjusted; model 2: age and body mass index were adjusted; model 3: age, body mass index, race, educational level and household size were adjusted. OR: Odds Ratio. Q1:<1.09 mIU/L, Q2: 1.09-1.57 mIU/L, Q3: 1.57-2.295 mIU/L, Q4:>2.295 mIU/L.

In the subgroup analyses stratified by BMI categories, a positive association was found between *H. pylori* seropositivity and TSH among normal (Q4 vs Q1: OR = 1.894; 95% CI, 1.109-3.235; P=0.019) and overweight (Q4 vs Q1: OR = 2.124; 95% CI, 1.047-4.308; P=0.037) subjects in model 2. However, a negative association was found between *H. pylori* seropositivity and TSH among obese subjects in model 2 (Q4 vs Q1: OR = 0.468; 95% CI, 0.248-0.886; P=0.020) and model 3 (Q4 vs Q1: OR = 0.429; 95% CI, 0.220-0.837; P=0.013). No statistically significant association was shown in any other model. Details are presented in **Table 4**.

**Table 4:** Association of thyroid stimulating hormone level with Helicobacter pylori seropositivity based on subgroup of BMI.

Subgroup	TSH	Model 1		Model 2		Model 3	
		OR (95% CI)	P value	OR (95% CI)	P value	OR (95% CI)	P value
Normal	Q1	Ref		Ref		Ref	
	Q2	1.701(0.881-3.285)	0.114	1.660(0.982-2.805)	0.059	1.686(0.969-2.933)	0.064
	Q3	2.017(1.003-4.056)	0.049	1.527(0.887-2.628)	0.127	1.377(0.777-2.442)	0.273
	Q4	1.906(0.963-3.772)	0.064	1.894(1.109-3.235)	0.019	1.659(0.942-2.920)	0.08
							
Overweight	Q1	Ref		Ref		Ref	
	Q2	1.122(0.672-1.873)	0.66	1.814(0.928-3.546)	0.081	1.981(0.979-4.010)	0.057
	Q3	1.056(0.639-1.746)	0.83	1.903(0.928-3.904)	0.079	1.924(0.915-4.043)	0.084
	Q4	1.456(0.880-2.409)	0.143	2.124(1.047-4.308)	0.037	1.845(0.885-3.846)	0.102
							
Obese	Q1	Ref		Ref		Ref	
	Q2	0.960(0.513-1.798)	0.899	0.724(0.382-1.373)	0.323	0.756(0.383-1.491)	0.42
	Q3	0.614(0.318-1.183)	0.145	0.693(0.377-1.276)	0.24	0.638(0.336-1.213)	0.17
	Q4	1.268(0.676-2.379)	0.459	0.468(0.248-0.886)	0.02	0.429(0.220-0.837)	0.013

Model 1: no covariates were adjusted; model 2: age and sex were adjusted; model 3: age, sex, race, educational level and household size were adjusted. OR: Odds Ratio. Q1:<1.09 mIU/L, Q2: 1.09-1.57 mIU/L, Q3: 1.57-2.295 mIU/L, Q4:>2.295 mIU/L.

In the subgroup analyses stratified by age, a positive association was observed between the *H. pylori* seropositivity and TSH among subjects aged over 60 years (Q4 vs Q1: OR = 1.999; 95% CI, 1.118-3.575; P=0.020); however, the TSH was not related to *H. pylori* seropositivity in other groups. Details are presented in **Table 5**.

**Table 5:** Association of thyroid stimulating hormone level with *Helicobacter pylori* seropositivity based on subgroup of age.

Subgroup	TSH	Model 1			Model 2			Model 3	
		OR (95% CI)	P value		OR (95% CI)	P value		OR (95% CI)	P value
**30-45 years**	Q1	Ref			Ref			Ref	
	Q2	1.194(0.661-2.155)	0.556		1.213(0.668-2.203)	0.527		1.126(0.594-2.131)	0.717
	Q3	1.082(0.602-1.946)	0.792		1.141(0.628-2.072)	0.665		1.091(0.580-2.055)	0.787
	Q4	1.578(0.831-2.994)	0.163		1.629(0.850-3.122)	0.141		1.196(0.593-2.409)	0.617
									
**45-60 years**	Q1	Ref			Ref			Ref	
	Q2	1.273(0.608-2.668)	0.522		1.161(0.546-2.465)	0.698		1.106(0.506-2.417)	0.801
	Q3	1.500(0.677-3.323)	0.318		1.310(0.579-2.962)	0.517		1.340(0.568-3.164)	0.504
	Q4	1.959(0.884-4.340)	0.097		1.720(0.763-3.878)	0.191		1.673(0.716-3.912)	0.235
									
**>60 years**	Q1	Ref			Ref			Ref	
	Q2	1.731(0.940-3.187)	0.078		1.783(0.964-3.297)	0.065		1.826(0.964-3.457)	0.065
	Q3	1.484(0.815-2.701)	0.197		1.525(0.834-2.787	0.17		1.277(0.681-2.395)	0.447
	Q4	1.939(1.090-3.450)	0.024		1.999(1.118-3.575)	0.02		1.789(0.977-3.276)	0.059

Model 1: no covariates were adjusted; model 2: sex and body mass index were adjusted; model 3: sex, body mass index, race, educational level and household size were adjusted. OR: Odds Ratio. Q1:<1.09 mIU/L, Q2: 1.09-1.57 mIU/L, Q3: 1.57-2.295 mIU/L, Q4:>2.295 mIU/L.

## 4 Discussion

In this study, we conducted observational association analyses between TSH and *H. pylori* infection in the NHANES 1999-2000 dataset. To the best of our knowledge, this study is the first to demonstrate that high TSH levels might be significantly induce an increased risk of *H. pylori* infection using the data from NHANES. NHANES is characterized by a rigorous sampling design from the national population of the United States, high-quality research measurement, and detailed quality control procedures (31).

The results of this study suggest that there is a positive relationship between *H. pylori* seropositivity and TSH. In stratified analyses, the adjusted association of serum TSH with *H. pylori* seropositivity was statistically significant in male not female, and age over 60 years. Different TSH levels represent different thyroid function. NHANES documents provide a normal TSH reference range of 0.34-5.6mIU/L according to manufacturer guidelines (39).

Houston Consensus on *H. pylori* infection stated that *H. pylori* testing be considered in patients treated with medications whose absorption is known to be impacted by infection, such as thyroxin (40). Therefore, there must be a correlation between *H. pylori* infection and thyroid function. The mechanism by which high TSH increases the risk of *H. pylori* infection remains to be elucidated in detail. Based on the evidence available to date, it was reported that half-emptying time for liquids correlated with TSH level (r = 0.83, P < 0.0001) in type 1 diabetic patients (41). A review was also reported that TSH influence gastrointestinal function (42). We can speculate that low gastric motility may in relation to TSH level, which may lead to the gastric environment favorable to the colonization by *H. pylori (* 43). Such changes could involve the microbiota imbalance in gastric mucosa. Zhang et al. has reported that remodeling of the gut microbiota structure could inhibit the occurrence of gastric antral inflammation and promote gastric motility (44).In addition, current data has clarified the association of obesity with GI motility disorders, which due to the decrease of ghrelin and imbalance of gut microbiota and gut-brain axis (45), all above may be an explanation of a negative association between *H. pylori* seropositivity and TSH levels in obese subjects, its own gastric motility disorder exceeds that caused by high TSH, which thus weakening the risk of TSH in *H. pylori* infection.

It was found that no significant differences in serum levels of TSH between lean and overweight/obese subjects (46). A mendelian randomization study has provided no evidence for a clinically relevant association between *H. pylori* and BMI/obesity (47). This was consistent with our results. The longer history of using birth control pills was strongly associated with hypothyroidism, especially for more than 10 years (39). Indeed, this may be an explanation of no significant association between *H. pylori* seropositivity and TSH levels in female. Due to the feature of NHANES database, this study failed to include data on contraceptive use, the persuasion of correlation between *H. pylori* seropositivity and TSH levels in female cohort was weak. Previous study has found that *H. pylori* seropositivity rates were higher in subjects with four or more household members (48, 49). This was consistent with our finding. The above results can prove that our cohort population has universal *H. pylori* epidemic characteristics, and the research results obtained under this cohort have strong persuasiveness. Overall, the current available data are limited, and further mechanistic studies are still necessary.

However, there are limitations to our studies. First, the data used in this study are not current because the date of serum TSH levels and *H. pylori* seropositivity is only provided in NHANES 1999-2000. Second, this study was designed as a cross-sectional study, which did not allow us to determine the causality between TSH levels and *H. pylori* seropositivity. Third, the bias caused by other potential confounding factors that did not be adjusted in this study, such as hypertension and diabetes, which may affect the results. At last, supplementing T3, T4 and serum iodine data may be more persuasive but the data is unavailable in this cycle.

## 5 Conclusion

In conclusion, circulating TSH levels were associated with the risk of *H. pylori* infection, especially among male, overweight and elderly adults. These people should focus on *H. pylori* screening because *H. pylori* is closely related to gastric cancer.

## Data availability statement

The original contributions presented in the study are included in the article/supplementary material. Further inquiries can be directed to the corresponding author.

## Ethics statement

Ethical review and approval was not required for the study on human participants in accordance with the local legislation and institutional requirements. Written informed consent for participation was not required for this study in accordance with the national legislation and the institutional requirements.

## Author contributions

YX designed the experiment and supervised the study. DL contributed to formal analysis, JW wrote the manuscript. YX reviewed and revised the manuscript. All authors read and approved the final manuscript.

## Funding

This study was supported by the National Natural Science Foundation of China [No.81970502, No.81860107, No.82060109] and the Science and Technology Project of Jiangxi Province [No.20203BBG73051, No.20201ZDG02007].

## Conflict of interest

The authors declare that the research was conducted in the absence of any commercial or financial relationships that could be construed as a potential conflict of interest.

## Publisher’s note

All claims expressed in this article are solely those of the authors and do not necessarily represent those of their affiliated organizations, or those of the publisher, the editors and the reviewers. Any product that may be evaluated in this article, or claim that may be made by its manufacturer, is not guaranteed or endorsed by the publisher.
